# The Impact of Energy Productivity and Eco-Innovation on Sustainable Environment in Emerging Seven (E-7) Countries: Does Institutional Quality Matter?

**DOI:** 10.3389/fpubh.2022.878243

**Published:** 2022-06-06

**Authors:** Adnan Safi, Yingying Chen, Liya Zheng

**Affiliations:** School of Economics, Qingdao University, Qingdao, China

**Keywords:** institutional quality, energy productivity, trade, carbon emission, eco-innovation, E-7 countries, public health, consumption-based carbon dioxide emission

## Abstract

Emerging economies are showing promising growth and economic success, but the growth process has significantly increased carbon emissions in these countries and deteriorated environmental quality. Environmental degradation is an issue of serious concern as it is directly linked to human lives and health. Since the creation of the Sustainable Development Goals (SDGs), the Emerging Seven (E-7) countries have struggled to meet the SDG targets, as it's been a challenge for them to lower carbon emissions and improve the quality of the environment. Thus, the present study explores the key factors that significantly affect environmental quality. This study examines the effect of institutional quality, energy productivity, and eco-innovation on consumption-based carbon dioxide (CCO_2_) emissions for E-7 economies. The cointegration analysis results show a long-run relationship between institutional quality, energy productivity, GDP, eco-innovation exports, imports, and CCO_2_ emissions. The results obtained using the cross-sectionally augmented autoregressive distributed lag (CS-ARDL) model show that institutional quality, energy productivity, eco-innovation, and exports adversely affect CCO_2_ emissions and improve environmental quality in the short and long run. In contrast, imports and GDP are positively linked with CCO_2_ emissions and contribute to environmental degradation. Policies that target institutional quality, eco-innovation, and energy productivity significantly affect CCO_2_ emissions and help improve environmental quality.

## Introduction

Climate change is an issue of serious concern for policymakers and researchers as it has a significant impact on human lives (1). Countries globally are taking steps to mitigate global warming since it had a critical impact on human lives and the environment through unexpected deviations in weather, melting of the glaciers, rising sea levels, and overall temperature. The key element that has a significant impact on global warming is greenhouse gas (GHG) emissions. Carbon emissions significantly contribute to environmental deterioration, accounting for 75% of GHG emissions (2, 3). For the purpose of addressing environmental deterioration, governments across the globe have proposed several accords, including the latest in 2015, the Paris Climate Accord, designed to control global warming to <2°C. Like the rest of the world, the Emerging-Seven countries have also set carbon neutrality goals, Brazil, and Mexico, have committed to achieve carbon neutrality (net-zero carbon emission) by 2050, and Turkey has set a target of 2053. Similarly, China, Russia, and Indonesia have set a target to attain carbon neutrality by 2060, whereas India has set a target of 2070 to achieve carbon neutrality. Numerous nations, including the emerging seven, are pressuring provinces, cities, and companies to accomplish net zero CO_2_ emissions and slash 80 to 100% of GHG emissions by 2050 (4).

In the Paris accord (COP-21), one of the important elements was the nationally determined contributions (NDCs) that required countries to establish national or domestic goals for carbon emission reduction. Countries worldwide, especially the E-7 countries, have committed to carbon neutrality. The majority of nations have set a target of zero emissions and carbon neutrality by 2,050–60; others, such as Uruguay and Norway, have set an even more challenging target to achieve net-zero carbon emission by 2030. Though, it is critical to reach the aims and objectives of net-zero carbon emissions and resolve climate change problems. Every country in the E-7 has established a goal for carbon neutrality. The E-7 nations are committed to attaining carbon neutrality by promoting zero net GHG emissions among organizations and localities. Due to its importance, numerous studies have been carried out to ascertain the key elements that can fasten the process of achieving the goal of zero carbon and identify factors that have a significant impact on environmental degradation. A substantial body of literature indicates that economic development is an essential predictor of environmental pollution (5–7). However, a limited but rising body of studies has been carried out on the linkage between institutional quality, energy productivity, eco-innovation, and the environment.

Previous studies have examined several possible variables in this respect, including political and economic effects. Though the results of these studies are not conclusive, Dal Bó and Rossi (8) suggested that increasing institutional quality might cut carbon emissions. The increase in resource allocation may also lower carbon emissions (9). However, Le et al. (11) noted that when a country lacks or has inadequate environmental legislation, some enterprises may regard it as the “pollution haven” and take chances to escape costly pollution control expenditures in other countries. Shah et al. (10) also suggested that the development process is degraded due to inadequate institutional quality that might raise risks and harm the environment. Though, a limited amount of empirical research has evaluated the influence of institutional quality and energy productivity on carbon emissions, with contradictory findings (10, 12). The empirical results continue to be conflicting (see, for example, (8, 9, 13–15). Furthermore, research studies on the dynamic link between energy productivity, institutional quality, and CO_−2_ emissions in the context of E-7 countries are limited (14). Institutions have been shown to influence economic growth (16), energy, and the environment (17–19). It is critical to include institutions in the productivity, economic growth, and CO_2_ emission nexus (20, 21).

Furthermore, previous research studies focus only on the influence of territory- or production-based CO_2_ emissions and have not taken into account the multinational manufacturing process. Previous studies have ignored the carbon emissions measure based on consumption that is adjusted for exports and imports. To put it another way, CO_2_ emissions are calculated using two different approaches: consumption-based carbon dioxide (CCO_2_) emissions and territory-based CO_2_ (TCO_2_) emissions. The standard metric is carbon emissions based on output, which excludes imports and exports. As a result, Peters et al. (22) developed a new database of CCO_2_ emissions, which is estimated as TCO_2_ emissions minus exports plus imports. Several research studies on both the CO_2_ data have shown different results for lower, middle- and high-income countries (23, 24).

Moreover, concerns have been expressed that high-income countries may reduce CO_2_ emissions through imports and exports by moving highly intensive CO_2_ emissions products to other countries. We estimated emission ratios based on consumption for the E-7 nations. The ratios for CCO_2_ and TCO_2_ are shown in Table 1. The findings of Table 1 indicate that China, Russia, India, and Indonesia, among the E-7 nations, are net carbon exporters, whereas Mexico, Turkey, and Brazil are net carbon importers. This means that China, Russia, India, and Indonesia export things that contribute to their decreased CO_2_ emissions, whereas Mexico, Turkey, and Brazil import products that increase their carbon consumption. This is because the E-7 nation's export and import of equipment and chemicals products decrease (increase) the country's CCO_2_ emissions^1^ (25).

**Table 1:** Ratios and difference for consumption and territory-based emissions.

**Country name**	**CCO_**2**_/TCO_**2**_**	**CCO_**2**_–TCO_**2**_**
Brazil	1.0107206	5.1853943
Mexico	1.0396787	18.06601
Turkey	1.0336859	14.12912
China	0.870745	−1330.027
Indonesia	0.96190721	−23.422668
India	0.905758	−245.011
Russia	0.83621317	−277.06946

*TCO_2_ represents territory-based CO_2_ emissions, and CCO_2_ shows consumption-based CCO_2_ emissions*.

Therefore, this research fills the gap by investigating the impact of eco-innovation, institutional quality, and energy productivity on CCO_2_ emissions for emerging-seven countries between 1995 and 2019. The current study fills the gap by adopting the recently established CCO_2_ emissions metric that considers fossil fuels and accounts for emissions embedded through exports and imports. Previous studies have used production or territorial-based carbon emissions and have overlooked the recently established CCO_2_ emissions. Thus, the present study fills the gap by investigating the influence of energy productivity, institutional quality, and eco-innovation on carbon emission. Additionally, previous search studies are mainly focused on developed countries; therefore, it's important to conduct this analysis for E-7 countries. The primary rationale for taking the E-7 economies is that they are large developing economies globally that have made tremendous strides over the last two decades. The gap between the E-7 and G-7 nations (United States of America, Canada, France, Japan, Germany, Italy, and the United Kingdom) is narrowing, and the E-7's economic growth may exceed that of the G-7 by 2032. Over the next 40 years, the E-7 Countries expected annual growth rate is projected to be 3.5 percent in comparison to 1.6 percent for the G-7 economies (26, 27). Additionally, the E-7 nations are big energy consumers, accounting for more than 40% of world energy consumption. As a result, it is critical to analyze the factors that contribute to CO_2_ emissions in the E-7 countries. Moreover, in this study, to determine the link between the variables, advanced econometric approaches are employed. In this study, to have a better outline of the influence of trade on CCO_2_ emissions, we analyzed trade by taking imports and exports as separate variables for the E-7 economies.

The rest of this paper is arranged as follows: the next section (section Literature Reviews) gives the literature review on institutional quality, eco-innovation, energy productivity, and carbon emission. Moreover, this part also gives the literature review for the control variables selected in this study (i.e., GDP imports and exports). The third part of this article discusses the research design, theoretical background, and analytical techniques employed in this study. Section Results explains and discusses the findings obtained using different statistical methods. The last part gives the conclusion and discusses policy implications.

## Literature Reviews

This section of the study discusses the existing literature on the factors that significantly affect CO_2_ emissions. Many scholars have examined the link between energy productivity, eco-innovation, institutional quality, economic development, international trade, and CO_2_ emissions [i.e., (12, 21, 25, 28–40)]. All these studies mentioned are discussed in this section below.

Economic activities have grown at an incredible rate in the last few decades, raising worries about their environmental effect. Increased economic activity results in an increase in people's income, but at the expense of natural resource depletion and environmental damage. Numerous studies have revealed substantial evidence of a unidirectional association between economic growth and carbon dioxide emission (35). Chang (41), for example, did research on China as a growing economy and discovered that the country's high CO_2_ emissions were a consequence of its economic expansion. Jardon et al. (36) conducted a research study taking data from 1971 to 2011 for 20 Latin American and Caribbean nations. The research revealed contradictory findings. When the cross-sectional dependency is neglected, the EKC hypothesis is verified; otherwise, it is rejected. Similarly, several studies have shown that economic growth is a key factor that enhances carbon emissions and deteriorates the environment (12, 25, 42).

Similarly, numerous studies have been carried out on the linkage between international trade and carbon emission. Isik et al. (37) argued that enhanced openness to global trade increases CO_2_ emissions for Greece. Likewise, Acheampong et al. (38) examine the influence of trade, renewable energy, and foreign direct investment on carbon emissions in Sub-Saharan Africa, demonstrating that trade contributes to carbon emissions. Additionally, several research studies have evaluated the linkage between trade openness and global carbon emissions. Stretesky and Lynch (39) evaluated the influence of exports on carbon emissions by taking panel data of 169 countries and concluded that exports would increase CO_2_ emissions. Acheampong et al. (38) and Stretesky and Lynch (39) employed the production-based carbon emission technique, which doesn't take into account international trade. In contrast to Stretesky and Lynch's (39) studies, Safi et al. (25) and Wahab et al. (12) studied the impact of international trade on carbon emission, taking CCO_2_ emissions as a measure that accounts for imports and exports to show that exports lessen CO_2_ emissions while imports boost CO_2_ emissions.

Studies examining the influence of eco-innovation on carbon emissions have shown that it improves environmental quality. The research of Zhang et al. (43) illustrates the relevance and influence of eco-innovation in reducing carbon emissions by taking China as a case study. Their study results showed energy efficiency and R&D as the main elements that minimize carbon emissions. For the period 1985–2012, in the case of Malaysia, Ali et al. (28) evaluated technological innovation as a determinant of carbon emission. According to the findings of the causality test, there is a two-way association among GDP and carbon emissions. Similarly, their results show that there is a similar linkage between technological innovation and CO_2_ emissions. On the other side, investments in sophisticated and environmentally friendly technology have been regarded as a means of decreasing CO_2_ dioxide emissions and improving the environment. Likewise, the research study of Ahmed et al. (44) showed that eco-innovation substantially enhances the environment by decreasing CO_2_ emissions by taking panel data from a sample of 24 European nations. This indicates that nations that implement clean technologies in their manufacturing processes may enhance the quality of their environment. Additionally, Mehsah et al. (45) studied the influence of innovation on carbon emissions in 28 OECD countries and demonstrated the validity of an inverted U-shaped association between carbon emission and innovation. This suggests that technological innovation is a key factor and enhances environmental quality in OECD nations in the long run.

Apart from the fact that all of the factors mentioned above have a substantial influence on carbon emissions, academics, economists, and regulators have given little consideration to the influence of energy productivity and institutional quality in the literature. Huaman and Jun (29) claim that increased energy production improves energy efficiency and, as a result, reduces environmental damage. Prior research has mostly focused on energy efficiency and intensity. The existing body of knowledge acknowledges that energy efficiency enhances environmental quality. In contrast, several research studies have utilized energy intensity as a proxy for a country's overall energy efficiency (46–48). In their study, Hasanbeigi et al. (47) argued that the primary drawbacks of taking energy intensity as a proxy for a country's energy productivity or efficiency are that the intensity rise may not always correspond to a real increase in the efficiency. For the reason that the existing research emphasizes primarily identifying the determinants of energy productivity, little is identified about energy productivity's ecological effect. As a result, a scant amount of research studies are focused on the environmental effect of energy productivity, and a recent study by Ding et al. (21) has shown that energy productivity may help mitigate CCO_2_ emissions taking G-7 nations as a case study. Similarly, Akram and Umar (32) showed that energy efficiency also decreases carbon emission and thus is one of the key factors that improve environmental quality.

After a thorough overview of the existing literature, it is obvious that scarce studies have studied the linkage between institutional quality and carbon emissions. Lau et al. (30) revealed the influence of institutional quality on the linkage between growth and carbon emissions taking Malaysia as a case study. The results suggested that unprejudiced and effective institutions are highly vital for economic advancement to minimize CO_2_ emissions. Ibrahim and Law (31) observed that institutional quality improves the environment and air quality. Moreover, international trade worsens the quality of air in countries with poor institutions as compared to countries with high institutional quality where trade improves air quality. Abid (17) included institutional quality in the debate between growth and emissions, taking data from 1990 to 2011 for 41 EU and 58 middle east, African (MEA) economies. He revealed that institutional quality is vital in the chosen nations for improving economic development and concurrently lowering CO_2_ emissions. Similarly, taking China as a case study, Ameer et al. (49) showed that institutional quality significantly decreases carbon emissions. In contrast, Azam et al. (50) have taken 66 developing nations as a case study to demonstrate that institutional quality enhances energy consumption and thus increases environmental degradation. Similarly, Godil et al. (33) study also showed that the country's economic growth and institutional quality enhance CO_2_ emissions by examining data from Pakistan from 1984 to 2018. Mehmood et al. (34) conducted a research study to determine the influence of institutional quality on CO_2_ emissions taking Pakistan, India, and Bangladesh as case studies from 1996 to 2016. The results were mixed and showed that In Bangladesh and India, the influence of institutional quality is negative on CO_2_ emissions, whereas in Pakistan, it raises CO_2_ emissions. It is clear from the above discussion that studies are inconclusive, and the studies mentioned above employed the territory-based carbon emission as a metric of environmental damage (i.e., CO_2_ emissions).

Unlike the studies discussed above, i.e., by Godil et al. (33), Jardon et al. (36), Azam et al. (50), and Akram and Umar (32), the current study fills the gap in the literature discussed and adds to it in many ways. Firstly, the present analysis uses the recently established CCO_2_ emissions metric that considers fossil fuels and accounts for emissions embedded through exports and imports. The studies discussed in the literature section [like (32, 33, 50)] have used production or territorial-based carbon emissions and have overlooked the recently established CCO_2_ emissions. Therefore, the present study fills the gap by investigating the effect of energy productivity, institutional quality, and eco-innovation on carbon emissions. Unlike earlier research investigations of Abid (17) and Zhang et al. (40), this study employs new econometric methods to evaluate the stationarity of the data, cointegration analysis, and long- and short-run estimates. In this study, we used the cointegration technique of Westerlund (51), Chudik et al.'s (52) CS-ARDL model to identify the link between institutional quality, energy productivity, eco-innovation, and CCO_2_ emissions. Moreover, we also evaluated the influence of E-7 countries' trade by analyzing imports and exports individually for a complete overview of the impact of trade on carbon emissions in E-7 nations.

## Methodology

This empirical study investigates the influence of institutional quality, eco-innovation, and energy productivity on CCO_2_ emissions taking economic growth, exports, and imports as control variables in the context of E-7 nations. Distinct from the prior research on CCO_2_ emissions [see, for instance, (12, 42, 53)], this research has taken different and unexplored exploratory variables of energy productivity, institutional quality, and eco-innovation. Moreover, we have applied advanced econometric methodologies to acquire the findings. Furthermore, the sample for this study is E-7 nations, and the time span is 1995 to 2018. The rationale for picking the time span of 1995–2018 is attributed to the data availability of the selected variables for E-7 countries. The dependent variable in this research is CCO_2_ emissions quantified in MtCO2e (Million tons) and is taken from the Global carbon atlas database created by Peters et al. (54). Economic growth data measured as the gross domestic product (GDP), imports (IM), and Exports (EX) is sourced from world development indicators (55). The data for eco-innovation or technological innovation (EcoInov) identified as the growth in environment-related technologies to the percentage of all technologies is obtained from the OECD database. To measure institutional quality in this study, we have developed an index based on the data collected from the world bank. We have taken six indicators to calculate the institutional quality of the E-7 economies, namely, voice and accountability, corruption control, political stability and the absence of violence/terrorism, the rule of law, regulatory quality, and government effectiveness. We built an aggregate index of these six factors stated above in order to have a cumulative score for assessing the institutional quality of E-7 countries.

### Theoretical Rationale

In this study, we examined the factors that significantly impact environmental pollution in the case of E-7 countries. The economic model for this study is developed following Safi et al. (25, 42, 56) and can be given as:


$$\begin{array}{l} CCO2_{i,t}=\vartheta_{0}+\vartheta_{1}InsQy_{i,t}+\vartheta_{2}\;EnPd_{i,t}+\vartheta_{3}GDP_{i,t}\\ \;+\vartheta_{4}\;EcoInov_{i,t}+\vartheta_{5}IM_{i,t}+\vartheta_{6}EX_{i,t}+\varepsilon_{i,t} \end{array}$$

In the above model, CCO_2_ stands for consumption-based carbon emissions, InsQy shows institutional quality, EnPd stands for energy productivity, GDP stands for economic growth, EcoInov stands for environmental-related technological innovation, IM stands for imports, EX stands for exports, ϑ_1_, ϑ_2_, ϑ_3_,ϑ_4_ ϑ_5_ and ϑ_6_ gives for parameters, and ε shows the error term. The logical reasoning for selecting the factors mentioned in the above equation Eq. (1) is in accordance with previous research with a strong theoretical motivation. Moreover, past research studies have only conducted studies based on the territory CO_2_ emissions, ignoring CCO_2_ emission, which is calculated as emissions within the boundaries of a country, also known as territorial-based CO_2_ emissions plus emissions from imports minus exports (57). Earlier research studies have ignored institutional quality and energy productivity for the E-7 group of countries, taking the recently established consumption-based emission metric; therefore, the present study fills the research gap. Institutions are mainly based on regulations, rules, and laws, which can act as an important medium to accomplish sustainable development goals. Additionally, the failure of institutions in a country can be harmful to the environment and leads to excessive emissions. Institutions in a country can help implement rules and regulations related to the environment, which can lower carbon emissions and improve environmental quality (31, 34). Similarly, countries with high institutional quality can boost their economy and keep in check high pollutant industries. Therefore, institutions play a vital role *via* rules, regulations, and laws implementation that ultimately affects carbon emission (17), thus $\vartheta_{1}=\frac{\vartheta_{\;}CC{O_{2}}_{i,t}}{\vartheta_{\;}InsQy_{i,t}}<0$. Based on studies (12, 21), we have taken energy productivity as the independent variable. Energy productivity has the potential to mitigate environmental degradation in the following ways. To begin, energy productivity results in a decrease in the import of fossil fuels, which results in a decrease in emissions. Additionally, it reduces energy expenses, and lastly, energy productivity reduces energy usage in the production of each unit, which ultimately improves environmental quality, therefore, $\vartheta_{2}=\frac{\vartheta_{\;}CC{O_{2}}_{i,t}}{\vartheta_{\;}EnPd_{i,t}}<0$. We also included economic growth as a control variable in this study based on the studies of (41, 58, 59). Increased economic activity enhances the demand and usage of energy, which is increased carbon emissions, which contributes to environmental degradation. Economic development is inextricably linked to energy consumption and is projected to have an effect on CCO_2_ emissions $\vartheta_{3}=\frac{\vartheta_{\;}CC{O_{2}}_{i,t}}{\vartheta_{\;}GDP_{i,t}}>0.$ We added imports as an independent variable in accordance with the studies of Safi et al. (42, 56) and Liddle (23). Goods produced in other nations and utilized in the E-7 are predicted to have a valuable influence on CCO_2_ emissions $\vartheta_{4}=\frac{\vartheta_{\;}CC{O_{2}}_{i,t}}{\vartheta_{\;}IM_{i,t}}>0$. Following Safi et al. (25) and Wahab et al. (12), we encompassed exports as an explanatory variable in our analyses. Exports help to decrease carbon emissions because they are associated with the use of sophisticated technology, which results in lower energy consumption, and because exported items are consumed in another nation, which also results in reduced energy consumption (60). Therefore, we predict that exports will have a negative effect on CCO_2_ emissions $\vartheta_{5}=\frac{\vartheta_{\;}CC{O_{2}}_{i,t}}{\vartheta_{\;}EX_{i,t}}<0$. Lastly, we have taken eco-innovation as an independent variable in this research. Eco-innovations have a detrimental effect on environmental deterioration and contribute to environmental quality improvement *via* a variety of routes. Eco-innovations have the potential to significantly improve business performance, cut energy consumption, and improve environmental quality. Eco-innovation minimizes carbon emissions associated with consumption *via* the use of environmentally friendly or innovative technology that results in fewer CO_2_ emissions. Thus, eco-innovation is anticipated to have an adverse effect on environmental degradation $\vartheta_{6}=\frac{\vartheta_{\;}CC{O_{2}}_{i,t}}{\vartheta_{\;}EcoInov_{i,t}}<0$. The predicted outcomes are as follows: ϑ_1_ < 0, ϑ_2_ < 0, ϑ_3_>0,ϑ_4_>0 ϑ_5_ < 0 and ϑ_6_ < 0.

### Analytical Framework

Prior to assessing the data's stationarity, we investigated slope heterogeneity and cross-sectional dependence. Both these tests are important in determining the accurate unit root test for this research study. Moreover, it's important to employ these tests and choose a unit root test that can account for cross-sectional dependence (CD) and slope heterogeneity (SH). We performed the method put forward by Pesaran and Yamagata (61) to test for SH and Pesaran's (62) technique to check for CD in the panel data. The slope homogeneity equation can be given as:


(2) $${\underline{\Delta }}_{SH}=\left(N\right)\frac{1}{2}\left(2k\right)-\frac{1}{2}\left(\frac{1}{N}\;\hat{S}-k\right)$$

(3) $${\underline{\Delta }}_{AdjSH}=\left(N\right)\frac{1}{2}\left(\frac{2k(T-k-1}{T+1}\right)-\frac{1}{2}\left(\frac{1}{N}\;\hat{S}-2k\right)$$

Where Δ_*SH*_ and Δ_*AdjSH*_ give the delta tilde and adjusted delta tilde, respectively.

After determining the SH and CD of the panel data, for unit root analysis, we adopted Pesaran's (62) cross-sectionally augmented IPS approach to determine the data stationarity we have gathered from various sources in this research study. The test used takes SH and CD into consideration. Additionally, the unit root results also provide drift and trend analysis. In this study, as cointegration analysis, we used Westerlund's (51) method to determine the link between institutional quality (InsQy), eco-innovation (EcoInov), and energy productivity (EnPd) and CCO_2_ emissions in the existence of imports, exports, and GDP in context of E-7 nations. The reason for selecting this technique is due to the fact that traditional data analysis approaches, such as random effects and fixed effects regression analysis, may provide inaccurate results because they cannot accurately express cross-sectional dependence in the error terms. Thus, we used the cointegration analysis technique established by Westerlund (51) to evaluate the linkage among the variables.

For our study to evaluate the short-run and long-run association between the selected variables, we used CS-ARDL (Cross-Section Augmented Auto-Regressive distrusted lag Model), the method put forward by Chudik et al. (52). The basic equation for CS-ARDL is as follows:


(4) $$\begin{array}{r} J_{i,t}=\;\Sigma_{I=0}^{p_{j}}\beta_{I,i}J_{i,t-I}+\Sigma_{I=0}^{p_{X}}\delta_{I,i}K_{i,t-I}\;+\;\in_{i,t} \end{array}$$

The above equation (4) gives the baseline equation for the CS-ARDL model; however, it does not address the problems of cross-section dependency, unobservable elements, non-stationarity, and slope heterogeneity. If we run the analysis in this equation, this will lead us to false and inaccurate results; therefore, the above equation is further extended to account for the factors mentioned above (52).


(5) $$\begin{array}{r} J_{it}=\;\Sigma_{I=0}^{p_{j}}\beta_{I,i},J_{i,t-I}+\Sigma_{I=0}^{p_{k}}\delta_{I,i}K_{i,t-I}+\Sigma_{I=0}^{p_{M}}{\sigma^{\prime }}_{i},I{\bar{M}}_{t-I}+\in_{i,t} \end{array}$$

In the above equation (5) ${\bar{M}}_{t-I}=\left(\bar{J_{i,t-I}},\bar{K_{i,t-I}}\right)$ gives us the averages, whereas the lags are shown using *p*_*j*_, *p*_*k*_, *p*_*M*_. The dependent variable is shown in Eq. (5) by *J*_*it*_ which is CCO_2_, the independent variables InsQy, EcoInov, EnPd, IM, EX, GDP are denoted by *K*_*i, t*_, for the trend or time $\bar{M}$ shows both cross-section and dummy averages. The long-run equation of CS-ARDL is as follows:


(6) $$\begin{array}{r} {\hat{\beta }}_{CS-ARDL,i}=\;\frac{\Sigma_{I=0}^{pK}{\hat{\delta }}_{I,i}}{1-\Sigma_{I=0}^{pJ}\hat{\beta_{I,i}}} \end{array}$$

The mean group can be given as:


(7) $$\begin{array}{r} {\hat{\bar{\beta }}}_{JG}=\;\Sigma_{i=1}^{N}\hat{\beta_{i}} \end{array}$$

In the same way, we derived short-run coefficients in equation (8):


$$\Delta J_{i,t}\;=\beta_{i}[J_{i,t-1}-\theta_{i}K_{i,t}]-\Sigma_{I=1}^{p_{J-1}}\beta_{I,i},\Delta_{I}J_{i,t-I}$$

(8) $$\begin{array}{r} +\Sigma_{I=0}^{p_{K}}\delta_{I,i}\Delta_{I}K_{i,t}+\Sigma_{I=0}^{p_{M}}{\sigma^{\prime }}_{i},I{\bar{M}}_{t}+\varepsilon_{i,t} \end{array}$$

(8.1) $$\begin{array}{r} \hat{\alpha_{i}}=-\left(1-\Sigma_{I=1}^{pJ}\hat{\beta_{I,i}}\right) \end{array}$$

(8.2) $$\begin{array}{r} {\hat{\delta }}_{i}=\;\frac{\Sigma_{I=0}^{pK}{\hat{\vartheta }}_{I,i}}{\hat{\alpha_{i}}} \end{array}$$

(8.3) $$\begin{array}{r} {\hat{\bar{\delta }}}_{JG}=\Sigma_{i=1}^{N}\hat{\delta_{i}} \end{array}$$

The long- and short-run estimations of the CS-ARDL model are given in equation (8), whereas ECM (-1) gives the speed of adjustment toward the equilibrium. The ECM values that are negative indicate convergence, whereas positive values of ECM indicate divergence. In addition, the outcome must be statistically significant. Additionally, to assess robustness, this analysis followed the AMG analysis (Augmented Mean Group) provided by the study of Eberhardt and Teal (63). Additionally, we used the Dumitrescu–Hurlin causality analysis to demonstrate a unidirectional relationship from institutional quality, eco-innovation, energy productivity, exports, economic growth, and imports to CCO_2_ emissions.

## Results

The outcomes of the econometric methodologies covered in section three are reported in this section. First, the outcomes of slope heterogeneity (SH) and cross-section dependency (CD) tests are given to determine the test to be conducted in this research study. Based on the CD and SH test results, we can decide on cointegration and unit root tests, as unit root tests and cointegration tests from the first generation can give us misleading outcomes. Thus, we first employed advanced robust econometric approaches that can cope with CD and SH's problems. We employed the Pesaran (64) CD test analysis, and the findings are given in the Table 2. The results of the CD test show that panels in our data are dependent cross-sectionally with significant results of test statistics, and the high value suggests shock in one nation impacts other countries.

**Table 2:** CD test analysis.

**Variables**	**Cd-stat**	**Meanabs**
CCO_2_	13.807***	0.562
GDP	22.072***	0.894
IMP	5.529***	0.452
EXP	6.996***	0.396
EnPd	10.259***	0.708
EcoInov	2.127***	0.218
InsQy	−2.661***	0.439

Asterisks *** show a 1% level of significance.

Similarly, model (1)-(3) gives the SH test results in Table 3. The findings of (1) to (3) are significant at a 1% significance level. Consequently, the null hypothesis of slope homogeneity is rejected. The findings of SH suggest that among the cross-sections, there are heterogeneity problems.

**Table 3:** Slope heterogeneity test.

**Models**	**Variables**	**Stat**	**Values**
Model 1	CCO_2_ GDP IMP EXP EcoInov	$\tilde{\Delta }$	9.772375***
		${\tilde{\Delta }}_{Adj}$	11.00957***
Model 2	CCO_2_ GDP IMP EXP EcoInov EnPd	$\tilde{\Delta }$	9.765034***
		${\tilde{\Delta }}_{Adj}$	11.25676***
Model 3	CCO_2_ GDP IMP EXP EcoInov EnPd InsQy	$\tilde{\Delta }$	9.417859***
		${\tilde{\Delta }}_{Adj}$	11.12092***

Asterisks *** show a 1% level of significance.

We employed Pesaran's (62) cross-sectionally augmented IPS method to test the stationarity of the data. Furthermore, the test findings incorporate the intercept and trend. The results for the unit root test are given in Table 4. The panel unit root results reveal that all indicators are stationary at 1st difference with the exception of CCO_2_ and eco-innovation (EcoInov), which were found significant at level. Following Safi et al. (25, 56) and Khan et al. (65), for the mix order stationarity, this study used the panel cointegration analysis of Westerlund (51) and the CS-ARDL econometric model that not only can deal with mix-order of integration but also accounts for slope heterogeneity and cross-sectional dependency of the panels.

**Table 4:** Unit root test.

**Variables**	**At level I (0)**		**At first difference, I (1)**	
	**Intercept**	**Intercept and**	**Intercept**	**Intercept and**
		**trend**		**trend**
CCO_2_	−3.457***	−3.296***	−4.368989***	−4.667316***
GDP	−1.373426	−1.210632	−2.934399***	−3.295852***
IMP	−1.922559	−2.478043	−4.542213***	−4.581494***
EXP	−1.573971	−2.281165	−4.367194***	−4.498726***
EcoInov	−3.965779***	−4.140779***	−5.844922***	−5.967767***
EnPd	−2.077191	−1.979793	−3.591024***	−4.016477***
InsQy	−1.049	−2.256349	−3.17351***	−3.296686***

Asterisks *** show a 1% level of significance.

Table 5 presents the results of the Westerlund cointegration analysis performed using the approach set out by Westerlund (51). The findings are given in the model (1)-(3), the mean group statistics are provided by Gt and Ga, and Pt and Pa provide the entire panel statistics. The findings for the models reveal a substantial long-run association between Institutional quality, eco-innovation, energy productivity, imports, exports, GDP, and CCO_2_ emissions at a 1 and 5% significance level.

**Table 5:** Panel cointegration test.

	**(1) CCO**_**2**_ **GDP**		**(2) CCO**_**2**_ **GDP IMP**		**(3) CCO**_**2**_ **GDP**	
	**IMP EXP**		**EXP Eco**		**IMP EXP EcoInov**	
	**EcoInov**		**Inov EnPd**		**EnPd InsQy**	
**Statistic**	**Value**	**Zt**	**Value**	**Zt**	**Value**	**Zt**
Gt	−7.803***	−14.983	−7.939***	−14.929	−7.051***	−11.994
Ga	−25.816***	−5.929	−25.710***	−4.819	−20.013**	−1.952
Pt	−25.984***	−17.1331	−24.771***	−16.196	−18.568***	−10.846
Pa	−29.858***	−8.5926	−27.206***	−6.379	−23.674***	−4.1293

Asterisks *** and ** shows 1 and 5% level of significance.

After verifying a long-run linkage amongst the variables, to assess the magnitude of the long and short-run connection for each factor, we employed the CS-ARDL approach. The findings in Table 6 reveal that all factors such as institutional quality (InsQy), Eco-innovation (EcoInov), energy productivity (EnPd), Imports (IM), and Exports (EX) and GDP have a substantial influence on CCO_2_ emissions. The result in model (3) reveals that in the short-run InsQy, EcoInov, EnPd, and EX have a substantial negative influence on CCO_2_ emissions with coefficients of −0.0061, −0.0282, −0.7486, and −0.3789, respectively. This suggests an increase in institutional quality, energy productivity, exports, and eco-innovation lower CO_2_ emissions in the E-7 group of countries. In contrast, economic growth and imports have a strong positive effect on CCO_2_ emissions with values of 0.5412 and 0.1142, respectively, showing an increase in GDP and imports boosts E-7 economies' CO_2_ emissions. Model (3) also gives the long-run coefficients for InsQy, EcoInov, EnPd, EX, IM and GDP that are −0.0041, −0.0320, −0.8806, −0.3895, 0.1189, and 0.5739, respectively.

**Table 6:** CS-ARDL.

	**(1)**	**(2)**	**(3)**
**Variables**	**CCO_**2**_**	**CCO_**2**_**	**CCO_**2**_**
ΔIMP	0.2466***	0.1155*	0.1142*
	(0.0608)	(0.0677)	(0.0569)
ΔEXP	−0.3528***	−0.2716***	−0.3789***
	(0.0983)	(0.0750)	(0.0906)
ΔGDP	0.5479***	0.4712***	0.5412***
	(0.1403)	(0.1132)	(0.1555)
ΔEcoInov	−0.0157*	−0.0342***	−0.0282*
	(0.0081)	(0.0099)	(0.0151)
ΔInsQy		−0.0029**	−0.0061**
		(0.0014)	(0.0030)
ΔEnPd			−0.7486***
			(0.2697)
ECM (-1)	−0.9123***	−0.8383***	−0.9984***
	(0.0473)	(0.0823)	(0.0819)
**Long-Run**			
GDP	0.6438***	0.6105***	0.5739***
	(0.2080)	(0.1721)	(0.1739)
EXP	−0.4043***	−0.3083***	−0.3895***
	(0.1176)	(0.0819)	(0.0964)
IMP	0.2735***	0.1758*	0.1189**
	(0.0692)	(0.0892)	(0.0588)
EcoInov	−0.0174*	−0.0384***	−0.0320*
	(0.0092)	(0.0109)	(0.0181)
InsQy		−0.0038**	−0.0041**
		(0.0018)	(0.0020)
EnPd			−0.8806**
			(0.4032)
F	1.5671**	2.7239***	1.6320**
CD-Stat	−1.7289*	−2.7497***	−1.7570*
	[0.0838]	[0.0060]	[0.0789]

The standard errors are in parentheses, in the CD stat row, the brackets [] give the p-values, and Asterisks *, **, *** show the significance level at 10, 5 and 1 percent.

The empirical results of institutional quality (InsQy) in Table 6 indicate a negative linkage with CCO_2_ emissions. Model (3) results show that InsQy causes a −0.0061 percent decrease in CCO_2_ emissions in the short term. This relationship is similar in the long term when InsQy results in an average decline of −0.0041percent in CCO_2_ emissions. The results revealed that the increase in institutional quality in both the short and long-run significantly reduces CCO_2_ emission in E-7 economies. The logical reasoning for that is that institutions are mainly based on regulations, rules, and laws, which can act as an important medium to achieve the goal of sustainable development goals. Institutions in a country can help implement rules and regulations related to the environment, which can lower CO_2_ emissions and improve environmental quality. Similarly, countries with high institutional quality can boost their economy and keep in check high pollutant industries. Therefore, institutions play a vital role *via* rules, regulations, and laws implementation that ultimately affects carbon emission. Thus increase in institutional quality enhances the quality of the environment, and these results are also in line with the findings of Ibrahim and Law (31) and Mehmood et al. (34). In contrast, these findings contradict the studies, i.e., (33) that argue that institutional quality leads to a rise in CO_2_ emission.

The results also show that eco-innovation negatively affects CCO_2_ emission both in the long and short run. Eco-innovation causes a −0.0282 percent decrease in CCO_2_ in the short term. This relationship is similar in the long term, where Eco-innovation results in an average decline of −0.0320 percent in CCO_2_. The results revealed that an increase in the use of environment-related technological innovation leads to a decrease in energy consumption and lower carbon emissions. Eco-innovations have a detrimental effect on environmental deterioration and contribute to environmental quality improvement *via* a variety of routes. Eco-innovations have the potential to significantly improve business performance, cut energy consumption, and improve environmental quality. Eco-innovation minimizes carbon emissions associated with consumption *via* the use of environmentally friendly or innovative technology that results in fewer CO_2_ emissions. These results are also in line with the previously published studies by Ali et al. (44), Zhang et al. (43), and Khan et al. (53).

The results in model (3) for energy productivity (EnPd) also show a significant linkage with CCO_2_ emissions both in the long and short-run estimation. Energy productivity causes a −0.7486 percent decrease in CCO_2_ in the short term. This relationship is higher in the long term, where EnPd results in an average decline of −0.8806 percent in CCO_2_ emissions. The results revealed that an increase in the energy efficiency of E-7 nations leads to a decrease in the usage of energy that lowers CO_2_ emissions. Energy Productivity results in a decrease in the import of fossil fuels, which results in a decrease in emissions. Second, it reduces energy expenses, and lastly, energy productivity reduces energy usage in the production of each unit, which ultimately improves environmental quality. Our findings are similar to previous studies (12, 21), which conducted a similar study for G-7 economies. Our findings are also in line with the study of Amin et al. (66), who conducted a study on N-11 economies showing that energy productivity significantly decreases CCO_2_ emissions.

The results for economic growth in the model (1) to (3) show that it positively affects carbon emission both in the long and short run. Model (3) results reveal that economic growth causes a 0.5412 percent rise in CCO_2_ in the short term. This relationship is a little higher in the long term when economic growth results in an average rise of 0.5739 percent in CCO_2_ emissions. Increased economic activity increases the demand and usage of energy, the outcome of which is increased carbon emissions, which contribute to environmental degradation. Economic growth is inextricably related to energy consumption and enhances CCO_2_ emissions. Our findings are similar to (35, 41, 59).

Lastly, imports and exports results are given in Table 6 from the model (1) to (3). Imports greatly boost the E-7 nations' CCO_2_ emissions. Import rises carbon emission by 0.1142 percent and 0.1189 percent, respectively, in the short and long term. The findings reveal that the E-7 nations import energy-intensive items. The point clarifies the results that commodities manufactured in other states, imported and used in the E-7 nations, affect consumption and, ultimately, carbon emission. By contrast, exports have a detrimental influence on carbon emissions, with a −0.3789 and −0.3895 percent decline in CCO_2_ is caused by exports of E-7 countries. since commodities factory-made in E-7 nations and exported and consumed in other nations help lower E-7 economies' CO_2_ emissions and enhances the quality of the environment. These findings are similar to that of previous studies (12, 56, 60).

We employed the AMG analysis to test for the robustness of the CS-ARDL results. Model (1) to (3) in Table 7 gives the detailed results of all the variables. The results in Model (3) show that eco-innovation, intuitional quality, energy productivity, and exports significantly decrease carbon emissions with the values of the coefficients −0.0832, 0.005, −0.731, and −0.346, respectively. In contrast to this, imports and economic growth enhance CCO_2_ emissions with coefficients of 0.173 and 0.791. These results also confirmed the results previously obtained using the CS-ARDL analysis and presented in Table 6.

**Table 7:** AMG analysis.

	**(1)**	**(2)**	**(3)**
**Variables**	**CCO_**2**_**	**CCO_**2**_**	**CCO_**2**_**
GDP	0.459***	0.600***	0.791***
	(0.056)	(0.093)	(0.111)
IMP	0.054	0.215**	0.173***
	(0.065)	(0.094)	(0.050)
EXP	−0.261***	−0.360***	−0.346***
	(0.058)	(0.062)	(0.046)
EcoInov	−0.0108*	−0.027***	−0.0832**
	(0.0057)	(0.002)	(0.0390)
InsQy		0.006*	0.005*
		(0.003)	(0.002)
EnPd			−0.731***
			(0.264)
constant	−30.977***	−31.869***	−47.370***
	(1.824)	(0.798)	(5.986)
Wald-Statistics	87.544	146.362	126.419

The standard errors are in parentheses, and asterisks *, **, *** show the significance level at 10, 5 and 1 percent.

The results of the Dumitrescu–Hurlin causality test are provided in Table 8. The results demonstrate the relationship from energy productivity, institutional quality, eco-innovation, GDP, imports, and exports to CCO_2_ emissions. The outcomes show that any policy targeting these aspects would significantly improve environmental quality. The E-7 may achieve sustainable development goals by focusing on these factors in order to decrease carbon emissions and enhance the quality of the environment.

**Table 8:** Causality analysis.

**Direction**	**W-bar**	**Z-bar stat**	***P* Value**
GDP → CCO_2_	4.818***	7.142	0.000
IMP → CCO_2_	4.183***	5.955	0.000
EXP → CCO_2_	5.058***	7.592	0.000
EcoInov → CCO_2_	4.784*	1.927	0.054
EnPd → CCO_2_	10.457***	6.040	0.000
InsQy → CCO_2_	8.897**	2.213	0.027

Asterisks *, **, *** show the significance level at 10, 5 and 1 percent.

## Conclusion and Policy Recommendations

Emerging economies are showing promising growth and economic success, but the growth process has increased these countries' carbon emissions. Since the creation of the Sustainable Development Goals (SDGs), the E-7 nations have struggled to meet the SDG targets, as it's been a challenge for them to lower CO_2_ emissions and enhance environmental quality. Several studies have been conducted to analyze the key factors that can reduce CO_2_ emissions in developing countries. In this context, the present study extends previous studies by exploring the impact of institutional quality, energy productivity, and eco-innovation on consumption-based carbon dioxide (CCO_2_) emissions in the presence of control variables GDP, imports, and exports. The cointegration analysis results showed a long-run connection among institutional quality, energy productivity, GDP, eco-innovation imports and exports, and CCO_2_. The results of the CS-ARDL analysis showed that institutional quality, energy productivity, eco-innovation, and exports have a significant adverse influence on CCO_2_ emissions and aid in enhancing environmental quality. In contrast to these results, imports and GDP showed that they are positively linked with CCO_2_ emissions and contribute to environmental degradation. The results obtained using AMG analysis as a robustness check further confirm these findings. The results using the causality test analysis demonstrate that policies that target eco-innovation, institutional quality, energy productivity, exports, GDP, and imports significantly affect carbon emissions and help in enhancing the quality of the environment.

According to the results of our study, policymakers in the E-7 nations should prioritize improving institutions in terms of implementing laws, rules, and regulations to have better implantation of the government's policy in these countries. It will improve not only economic growth but also enhance environmental quality and help achieve sustainable development goals set by E-7 countries. The results of this study suggest E-7 countries should adopt eco-friendly technology that will help significantly reduce environmental degradation and improve economic development in the E-7 countries. Additionally, E-7 countries should focus on enhancing their industry efficiency and energy productivity, as an increase in energy productivity will lower the demand for energy and energy consumption, ultimately leading to low carbon emissions. This will improve environmental quality and help E-7 economies achieve sustainable development goals. According to the conclusions of this research, the E-7 nations should impose environmental levies to incentivize businesses to evade energy-intensive products in favor of clean, green energy efficient renewable alternatives. Additionally, the causality analysis results demonstrate that policies targeting institutional quality, eco-innovation, energy productivity, exports, GDP, and imports significantly affect CCO_2_ emissions and help improve environmental quality. Institutional quality, eco-innovation, and energy productivity will greatly cut CO_2_ emissions and help achieve the E-7 nations' aim of sustainable development goals.

## Data Availability Statement

The original contributions presented in the study are included in the article/supplementary materials, further inquiries can be directed to the corresponding author.

## Author Contributions

AS: conceptualization, methodology, software, validation, data curation, writing, review, and original draft. YC: supervision, resources, review, and editing. LZ: methodology, validation, writing, review, and editing. All authors contributed to the article and approved the submitted version.

## Conflict of Interest

The authors declare that the research was conducted in the absence of any commercial or financial relationships that could be construed as a potential conflict of interest.

## Publisher's Note

All claims expressed in this article are solely those of the authors and do not necessarily represent those of their affiliated organizations, or those of the publisher, the editors and the reviewers. Any product that may be evaluated in this article, or claim that may be made by its manufacturer, is not guaranteed or endorsed by the publisher.

## Footnotes

^1^The import and export of E-7 economies in the last five years reveals that China exported 49% machines, India exported 14% chemical products, Indonesia exported 23% mineral products Russia exports comprised 28% crude oil, Brazil imports comprised 24% machinery, and 21% chemicals, Mexico imports comprised 37% machinery and 27% transportation, Turkey imports comprised 23% machines and 14% metals (67). According to these numbers, the majority exported items by carbon exporters and imported by carbon importers in the E-7 countries are equipment, chemicals, mineral, and transportation.
